# Disease-driven restructuring of the gut microbiome underlies inflammatory bowel disease dysbiosis

**DOI:** 10.3389/fmicb.2025.1744574

**Published:** 2026-01-27

**Authors:** Zixu Ding, Ke Ren, Yixue Xu, Tong Feng, Kuiqing Cui, Qingyou Liu, Cun Liao

**Affiliations:** 1 Department of Colorectal and Anal Surgery, The First Affiliated Hospital of Guangxi Medical University, Nanning, China; 2 Guangdong Provincial Key Laboratory of Animal Nutrition Control, College of Animal Science, South China Agricultural University, Guangzhou, Guangdong, China; 3 College of Basic Medical Sciences, Zhejiang Chinese Medical University, Hangzhou, China; 4 State Key Laboratory for Conservation and Utilization of Subtropical Agro-Bioresources, College of Animal Science and Technology, Guangxi University, Nanning, China; 5 Department of Bioinformatics and Systems Biology, Key Laboratory of Molecular Biophysics of the Ministry of Education, Hubei Key Laboratory of Bioinformatics and Molecular Imaging, Center for Artificial Biology, College of Life Science and Technology, Huazhong University of Science and Technology, Wuhan, China; 6 Provincial Key Laboratory of Animal Molecular Design and Precise Breeding, School of Life Science and Engineering, Foshan University, Foshan, China

**Keywords:** dysbiosis, ecological network, gut microbiota, inflammatory bowel, metagenomics

## Abstract

**Background:**

Inflammatory bowel disease (IBD) is a chronic and recurrent intestinal disorder with rising global incidence, yet its complex pathogenesis remains poorly understood, underscoring the need to clarify the microbial mechanisms underlying intestinal inflammation. IBD is associated with a profound imbalance of the gut microbial ecosystem. However, the ecological and functional remodeling of the gut microbiota during IBD progression remains unclear. This study used metagenomic sequencing to investigate microbial composition, functional capacity, and ecological interactions in the gut microbiota of IBD patients compared with healthy individuals.

**Results:**

The IBD group exhibited significantly reduced microbial diversity and a distinct community structure compared with healthy controls. Pro-inflammatory genera such as g_*Fusobacterium* (*p* < 0.001) and g_*Morganella* (*p* < 0.001) were enriched, whereas short-chain fatty acid producing bacteria, including g_*Ruminococcus* (*p* < 0.0001) and g_*Agathobacter* (*p* < 0.0001), were markedly depleted. Functional annotation revealed decreased abundance of carbohydrate-active enzymes (GH3, GH44, GH53, and GH77; all *p* < 0.05) associated with polysaccharide degradation, together with enrichment of pathways related to immune activation and inflammation, such as the JAK–STAT and chemokine signaling pathways (*p* < 0.05). Co-occurrence network analysis further showed that IBD-associated microbes formed positively correlated clusters dominated by inflammatory taxa, whereas healthy microbiota were organized around SCFA-producing commensals.

**Conclusion:**

Compared with healthy individuals, the gut microbiota of IBD patients undergoes functional reprogramming characterized by loss of metabolic versatility and enrichment of inflammation-related pathways. These findings provide new insights into the ecological and metabolic mechanisms through which the gut microbiota contribute to intestinal inflammation and disease progression.

## Introduction

Inflammatory bowel disease (IBD), a chronic and relapsing inflammatory disorder of the gastrointestinal tract, has become a growing global health concern as its incidence continues to rise beyond traditionally high-prevalence regions (Bisgaard et al., 2022). According to the Lancet Global Burden of Disease study, IBD exhibits a clear geographic transition, with incidence rates stabilizing in traditionally high-prevalence regions while increasing markedly in newly industrialized countries such as China, which are emerging as new disease hotspots (Jairath and Feagan, 2020). This epidemiological shift is closely linked to environmental and lifestyle factors, including rapid urbanization, Westernization of dietary patterns, and reduced exposure to environmental microorganisms (Ward and Goldie, 2024). From a burden-of-disease perspective, inflammatory bowel disease imposes a significant dual burden on healthcare systems and the broader economy, particularly in middle- and high-income countries, where direct medical costs can consume a substantial portion of household income (Wang et al., 2023). Given its escalating prevalence and significant health and economic impacts, elucidating the pathogenic mechanisms of IBD and developing novel preventive and therapeutic strategies have become urgent priorities in both scientific research and clinical practice.

In recent years, the gut microbiota has emerged as a central regulator in the pathogenesis of inflammatory bowel disease (IBD), serving as a critical interface between the external environment and the host immune system (Shah and Itzkowitz, 2022). Rather than a passive bystander, the intestinal microbiome constitutes an active and dynamic ecosystem that plays a vital role in maintaining mucosal and immune homeostasis (Banerjee et al., 2020). In healthy individuals, commensal microorganisms contribute to intestinal stability through multiple metabolic and immunoregulatory mechanisms (Ma et al., 2022). For instance, short-chain fatty acids (SCFAs) such as butyrate, produced by microbial fermentation of dietary fibers, provide an essential energy source for colonocytes and modulate the differentiation and function of regulatory T cells (Tregs) via epigenetic regulation, including inhibition of histone deacetylases (Krause et al., 2024). Similarly, microbial metabolites derived from tryptophan metabolism and secondary bile acid transformation act as signaling molecules that promote intestinal immune tolerance through activation of the aryl hydrocarbon receptor (AhR) and other pathways (Michaudel et al., 2023).

Disruption of tuned microecological balance is a hallmark of IBD (Ning et al., 2023). Numerous studies have demonstrated that patients with IBD exhibit decreased microbial diversity, increased community heterogeneity, and marked shifts in microbial composition, characterized by depletion of beneficial commensals and overrepresentation of potentially pathogenic taxa (Cannarozzi et al., 2024; Valdés-Mas et al., 2025). These alterations are frequently accompanied by functional impairments in microbial metabolism, including reduced capacity for nutrient degradation and compromised mucosal barrier integrity (Mills et al., 2022). Such findings underscore the pivotal role of gut microbial dysbiosis in the initiation and progression of IBD, highlighting the need for a deeper understanding of the microbiome-driven mechanisms underlying intestinal inflammation. *Fusobacterium nucleatum* has been shown to aggravate intestinal inflammation by upregulating CARD3 and activating IL-17F-mediated NF-κB signaling, resulting in elevated pro-inflammatory cytokines in the gut mucosa (Zheng et al., 2025). Pathogenic *Proteobacteria*, particularly adherent-invasive *Escherichia coli*, are associated with adhesion to and invasion of intestinal epithelial cells, triggering increased TNF-α and IL-8 production and sustained immune activation (Palmela et al., 2018). Additionally, enrichment of pro-inflammatory taxa such as *Ruminococcus gnavus* has been reported in IBD and correlates with elevated inflammatory responses, underlining the role of dysbiotic pathobionts in promoting intestinal inflammation (Shah et al., 2025).

Despite extensive research revealing structural dysbiosis of the gut microbiota in IBD patients, current understanding remains largely descriptive, focusing on compositional differences rather than providing a systematic characterization of functional remodeling (Iliev et al., 2025; Yuan et al., 2021). In particular, how the evolution of carbohydrate-active enzyme (CAZyme) repertoires shapes microbial metabolic strategies and ecological interactions, thereby reshaping the host–microbiota metabolic interface, has long been overlooked (Labourel et al., 2023). Moreover, the relative contributions of environmental factors, including disease status, dietary patterns, and host characteristics, to microbial structural and functional variations remain insufficiently explored (Piovani et al., 2019).

To systematically dissect the ecological and functional alterations of the gut microbiome in IBD, we performed metagenomic sequencing of fecal samples from 40 patients and 47 healthy controls. The analysis revealed a distinct microbial imbalance, featuring the enrichment of pro-inflammatory taxa and depletion of fiber-degrading commensals, accompanied by a shift in metabolic potential toward inflammation-adaptive pathways. Network-based and statistical modeling further indicated that disease status plays a predominant role in driving microbial community restructuring. Together, these findings establish an integrative framework linking microbial dysbiosis, functional remodeling, and host–environment interactions, providing a systems-level perspective for developing microbiome-based diagnostic and therapeutic strategies in IBD.

## Materials and methods

### Sample collection

A total of 40 patients with inflammatory bowel disease (IBD) and 47 healthy family members living in the same households and sharing similar lifestyle and dietary habits were recruited. The IBD cohort consisted of 27 Crohn’s disease (CD) patients and 13 ulcerative colitis (UC) patients, diagnosed according to the Montreal Classification (Supplementary Table S1). None of the participants had received antibiotics within the 2 months prior to enrollment, and individuals with other intestinal disorders were excluded. Fecal samples were collected from the midstream of naturally passed stool, placed in sterile ice boxes, and transferred into 2 mL centrifuge tubes within 30 min. Samples were subsequently stored at −80 °C until further analysis. The study protocol was approved by the Ethics Committee of our institution, and written informed consent was obtained from all participants.

### DNA extraction, metagenomic sequencing, and data processing

Genomic DNA was extracted from approximately 200 mg of each fecal sample using the QIAamp PowerFecal Pro DNA Kit (Qiagen, Hilden, Germany, Cat. No. 51804) according to the manufacturer’s instructions. To ensure efficient lysis of a wide range of microorganisms, including tough-to-lyse Gram-positive bacteria, the protocol incorporated a bead-beating step with a mixture of 0.1 mm and 0.5 mm zirconia/silica beads. The extracted DNA was eluted in 50 μL of TE buffer (10 mM Tris–HCl, 1 mM EDTA, pH 8.0). DNA concentration and purity were assessed using a Qubit® 2.0 Fluorometer (Thermo Fisher Scientific, USA) and a NanoPhotometer® N60 (Implen, Germany; acceptable A260/A280 ratio > 1.8), respectively. The integrity of the DNA was checked by 1% agarose gel electrophoresis to confirm the presence of high-molecular-weight fragments (Ding et al., 2025). All qualified DNA samples were stored at −80 °C until library preparation.

Metagenomic libraries were constructed from 500 ng of high-quality DNA using the Illumina TruSeq DNA PCR-Free Library Preparation Kit (Illumina, USA) according to the manufacturer’s guidelines, yielding an average insert size of approximately 550 bp. The libraries were sequenced on an Illumina NovaSeq 6000 platform (Illumina, USA) to generate 2 × 150 bp paired-end reads, with a target of >85% of bases achieving a Q-score ≥ 30. An average of 10 Gb of raw data was generated per sample.

Raw sequencing reads were processed through a bioinformatic pipeline as follows: (1) Adapter sequences and low-quality bases were trimmed using Trimmomatic v0.35 with parameters: ILLUMINACLIP:TruSeq3-PE.fa:2:30:10 SLIDINGWINDOW:4:20 MINLEN:110 (Bolger et al., 2014). (2) To eliminate host contamination, quality-filtered reads were aligned to the human reference genome (GRCh38) using Bowtie2 v2.3.3 in—very-sensitive-local mode (Qin et al., 2010). The aligned reads (identified as host-derived) were discarded, resulting in an average of 0.5% of reads being removed per sample. The remaining high-quality, host-free reads were used for downstream analysis.

### Taxonomic and functional annotation of human gut microbiome

Taxonomic and functional profiling were performed directly on the high-quality, host-free reads using the Unified Human Gastrointestinal Genome (UHGG v1.0) catalog and its associated protein database (Almeida et al., 2021). For taxonomic composition, strain-level relative abundances were quantified by aligning reads to the UHGG representative genomes using Salmon v1.10.0 (Patro et al., 2017) in alignment-based mode, with the resulting abundances being aggregated to generate profiles at higher taxonomic ranks (genus, family, phylum). Concurrently, the functional potential was characterized by aligning the same set of reads to the UHGG protein catalog using HUMAnN3 v3.7 (Beghini et al., 2021), which quantifies gene family abundance and functionally stratifies them into KEGG Orthology (KO) groups, KEGG pathways (v2023), Clusters of Orthologous Groups (COG) categories (Galperin et al., 2015; Huerta-Cepas et al., 2016), and Carbohydrate-Active Enzymes (CAZymes v9.0) via integrated annotation against the CAZy database (Zhang et al., 2018). Finally, differential abundance of microbial taxa between pre-defined groups was identified using LEfSe (Linear Discriminant Analysis Effect Size), applying a Wilcoxon rank-sum test (*α* = 0.05) and a logarithmic LDA score threshold of >2 (Cao et al., 2023).

### Statistical analysis using linear mixed-effects models

To investigate the effects of host and environmental factors on gut microbial composition at the genus level, we employed linear mixed-effects models (LMMs) (Luke, 2017). Prior to modeling, raw microbial abundance data were preprocessed to ensure consistency and comparability: (1) genus-level counts were extracted from the metagenomic dataset, transposed such that samples corresponded to rows and genera to columns, and converted to numeric values; (2) taxa with zero counts across all samples were removed; (3) cumulative sum scaling (CSS) normalization was applied to adjust for varying library sizes (Paulson et al., 2013); (4) All genera were included in the downstream statistical analysis to comprehensively assess associations with host and environmental factors. Environmental and host metadata, including disease status, age, diet type, and gender were matched to microbiome samples, and only samples present in both datasets were retained. All categorical variables were coded as factors.

For each genus, we fitted a linear mixed-effects model of the form:


$$Y_{\mathrm{ij}}=\beta_{0}+\beta_{1}\text{DiseaseStatu}s_{i}+\beta_{2}\mathrm{Ag}e_{i}+\beta_{3}\text{DietTyp}e_{i}+\beta_{4}\text{Gende}r_{i}+\varepsilon_{\mathrm{ij}}$$

where $Y_{\mathrm{ij}}$ represents the normalized abundance of genus *j* in sample *i*, *β* denotes fixed-effect coefficients, and $\varepsilon_{\mathrm{ij}}$ is the residual error. Random effects could be incorporated if hierarchical structure or repeated measurements were present; however, all samples were independent, so we primarily used fixed-effects models. Model fitting was performed using the lme4 R package, and significance testing for fixed effects was conducted using the lmerTest package (Lee and Braun, 2012).

Variance components were calculated from the ANOVA decomposition of each model to quantify the proportion of variance explained by each factor. The total model explanatory power (*R*^2^) was reported for each genus. To visually summarize the results, bar plots of variance contributions, heatmaps of factor effects, and network diagrams linking environmental variables to genera with substantial explained variance were generated using ggplot2, reshape2, and igraph/ggraph packages (Mora and Donaldson, 2011).

Model diagnostics, including residual plots, Q–Q plots, and coefficient confidence intervals, were assessed to ensure model validity. Additionally, for genera most strongly associated with disease status, effect plots and coefficient plots were generated using the effects package to visualize the direction and magnitude of each factor’s influence. All analyses were performed in R (version 4.5.1). A random seed was set to 123 at the outset to ensure the reproducibility of all statistical results.

### Statistical analysis of microbiome-environment associations

To disentangle the complex interactions between microbial genera and environmental variables, we implemented an integrated correlation–network analysis (Faust and Raes, 2012). For each genus–environment pair, the association strength was quantified as the proportion of variance in genus abundance explained by the environmental factor (contribution%), derived from the linear mixed-effect models described above. A bipartite network was subsequently constructed, in which microbial genera and environmental factors were represented as nodes, and edges were established when the explained variance exceeded 1% (Wen et al., 2025). The edge weight corresponded to the magnitude of variance explained, encoding the strength of each association. Network visualization and topological analysis were performed using the igraph and ggraph packages in R (Hickey et al., 2024). Key topological parameters, including the number of nodes, number of edges, network density, average degree, and modularity index, were calculated to characterize network complexity and structural organization. The robustness of the network topology was further evaluated by testing multiple contribution thresholds, and the resulting topological parameters are summarized in the Results section and corresponding Supplementary figures.

### Statistical analysis

All statistical analyses were performed using R software (version 4.5.1) and SPSS (version 27.0). Data are presented as mean ± standard deviation (SD) for normally distributed variables. Differences between groups were assessed using Student’s t-test, as implemented in GraphPad Prism (version 9.0). Statistical significance was defined as *p* ≤ 0.05 (*), *p* ≤ 0.01 (**), *p* ≤ 0.001 (***), and *p* ≤ 0.0001 (****).

## Results

### Significant differences in gut microbial community structure between healthy individuals and IBD patients

The gut microbial community structure differed markedly between healthy individuals and patients with inflammatory bowel disease. To systematically compare the microbial features of these two populations, we collected fecal samples from 47 healthy controls and 40 IBD patients for metagenomic sequencing (Supplementary Figure S1).

**Figure 1:**
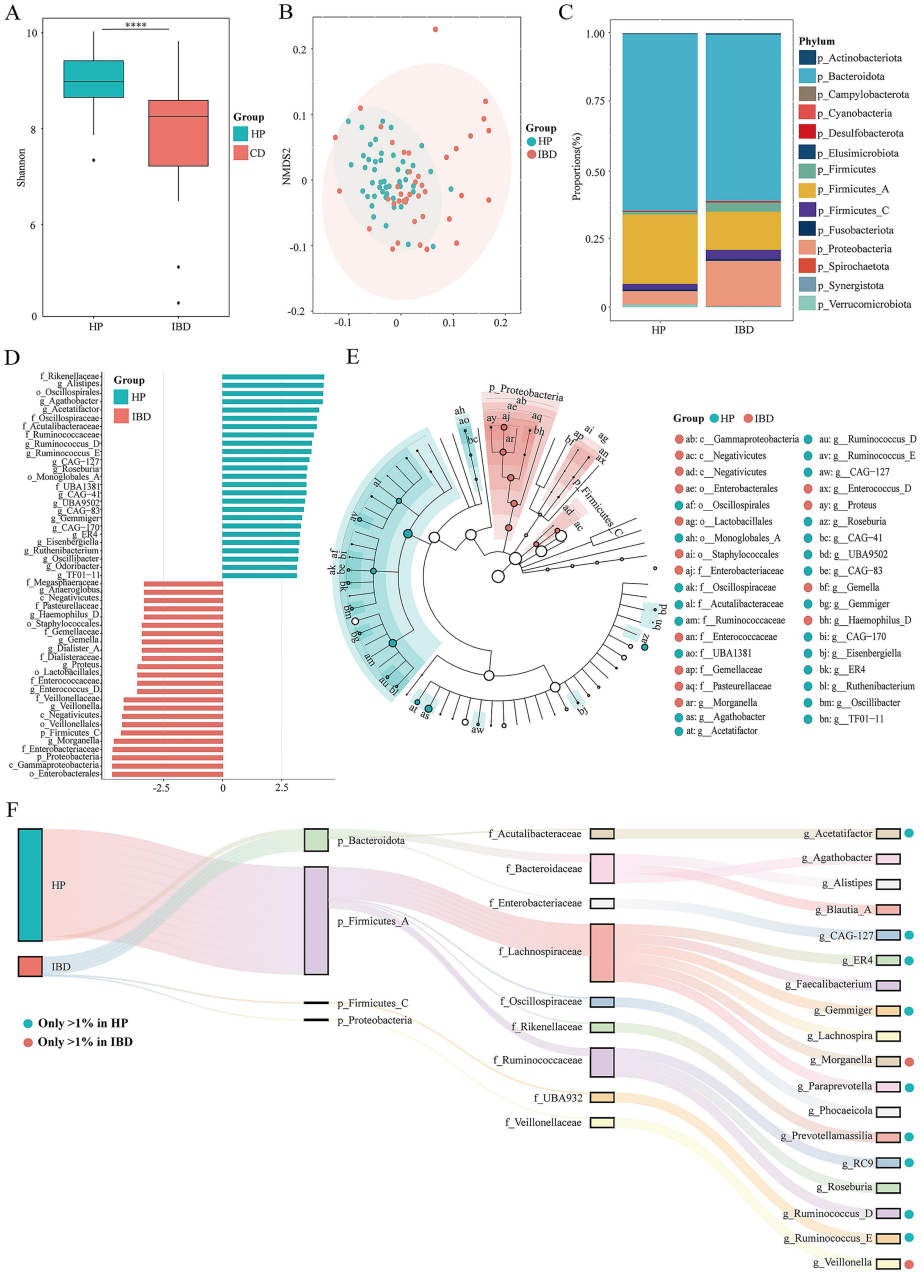
Microbial composition of the rumen in healthy individuals HP (*n* = 47) and patients with IBD (*n* = 40). **(A)** Alpha-diversity of rumen microorganisms in the HP and IBD groups. **(B)** Beta-diversity analysis of rumen microorganisms in the HP and IBD groups, visualized using NMDS. **(C)** Stacked bar plots showing the relative abundance of microbial phyla in the HP and IBD groups. **(D)** Histogram of LDA scores indicating differentially abundant microorganisms between the HP and IBD groups (LDA score > 2, *p* < 0.05). The length of each bar represents the effect size of significantly different taxa. **(E)** Cladogram illustrating phylogenetic distribution of differentially abundant taxa. Circles from inner to outer rings represent taxonomic levels from phylum to genus. Node size corresponds to relative abundance; nodes in purple and yellow denote taxa significantly associated with the HP and IBD groups, respectively. Taxa without significant differences are shown in white. **(F)** Sankey diagram depicting the flow and proportional contribution of microbial taxa from the phylum to genus level between the HP and IBD groups.

Alpha- and beta-diversity analyses revealed that the gut microbiota of healthy controls exhibited significantly higher α-diversity than that of IBD patients (*p* < 0.001; Figure 1A). Beta-diversity analysis further demonstrated a clear separation between the two groups (Figure 1B). Notably, the IBD samples were more dispersed in the β-diversity space, whereas samples from healthy individuals clustered more tightly, indicating greater interindividual heterogeneity in the gut microbiota of IBD patients.

At the phylum level (Figure 1C), p_Bacteroidota and p_Firmicutes were dominant in both groups. Compared with the healthy controls, IBD patients exhibited a marked increase in the relative abundance of p_Proteobacteria (HP: 4.83%, IBD: 16.53%), p_Firmicutes (HP: 0.95%, IBD: 3.34%), and p_Firmicutes_C (HP: 2.04%, IBD: 3.32%). In contrast, the relative abundance of p_Firmicutes_A (HP: 25.33%, IBD: 13.96%), p_Bacteroidota (HP: 64.43%, IBD: 60.77%), and p_Verrucomicrobiota (HP: 1.04%, IBD: 0.44%) was significantly reduced in IBD patients. p_Proteobacteria is widely recognized as a hallmark of dysbiosis and mucosal inflammation (Umeda et al., 2023), whereas the depletion of p_Bacteroidota and p_Verrucomicrobiota, key contributors to short-chain fatty acid production and epithelial homeostasis, indicates a loss of beneficial, symbiotic functions (Jia et al., 2024). To further identify differentially enriched taxa and potential microbial biomarkers, we performed linear discriminant analysis (LDA) and LEfSe analysis. In total, 37 taxa met the significance threshold (|LDA| ≥ 2.5, *p* < 0.05; Figure 1D). The cladogram generated by LEfSe (Figure 1E) revealed differential taxa from the phylum to family levels. The healthy group was characterized by enrichment of f_Oscillospiraceae, f_Acutalibacteraceae, and f_Ruminococcaceae, families associated with butyrate production and anti-inflammatory activity (Raygoza Garay et al., 2023), whereas f_Enterobacteriaceae, f_Enterococcaceae, f_Gemellaceae, and f_Pasteurellaceae commonly linked to inflammation and opportunistic infection were significantly enriched in the IBD group (Luo et al., 2023). At the genus level, genera with relative abundance greater than 1% were defined as dominant taxa. Dominant genera in the healthy group included *g_Acetatifactor*, *g_Gemmiger*, *g_Paraprevotella*, *g_Prevotellamassilia*, *g_Ruminococcus_D*, and *g_Ruminococcus_E*. In contrast, only *g_Morganella* and *g_Veillonella* were identified as dominant in the IBD group (Figure 1F).

Collectively, these findings demonstrate that the gut microbiota of IBD patients exhibits a dysbiotic state characterized by loss of microbial diversity, disrupted community structure, and expansion of potentially pro-inflammatory taxa.

### Dysbiosis characterized by depletion of scfa-producing bacteria and enrichment of pro-inflammatory taxa shapes the microbial signature of IBD

**Figure 2:**
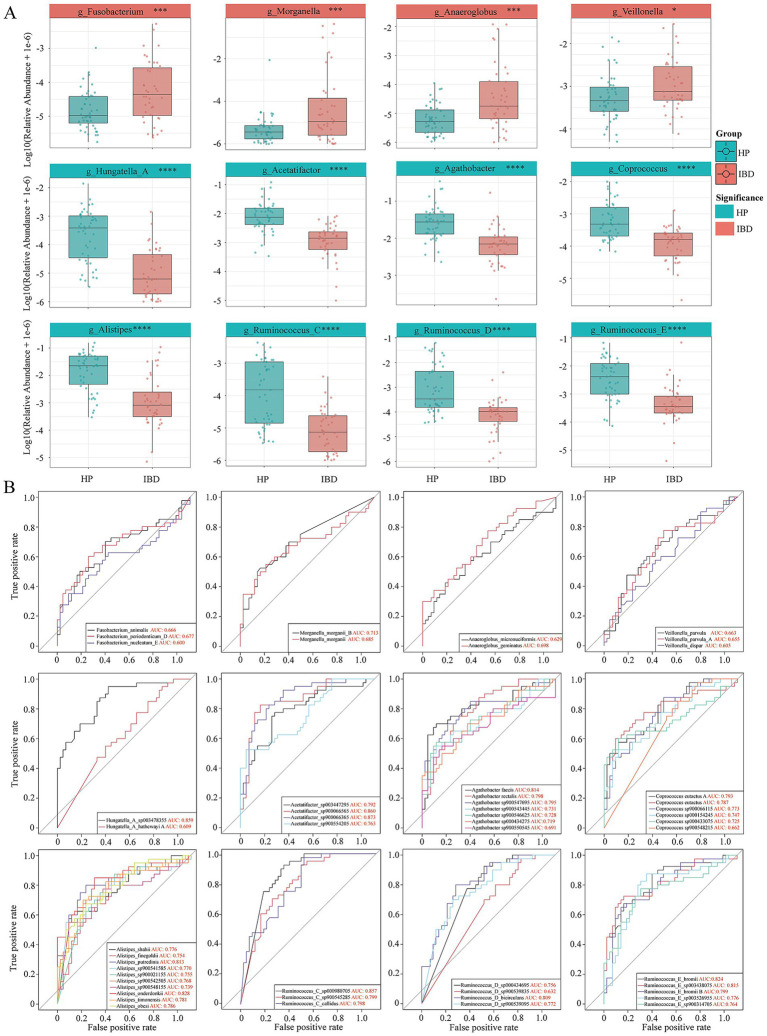
Differential genera and ROC analysis of microbial markers between healthy individuals (HP, *n* = 47) and patients with IBD (*n* = 40). **(A)** Box plots showing the relative abundance of significantly different genera between the HP and IBD groups. The statistically significant marker ** represents * for *p* ≤ 0.05; ** for *p* ≤ 0.01; and *** for *p* ≤ 0.001, *** for *p* ≤ 0.0001. **(B)** Receiver operating characteristic (ROC) curves evaluating the diagnostic potential of all significantly different bacterial species under the differential genera. The area under the curve (AUC) values reflect the discriminatory power of each species as a potential biomarker.

To identify bacterial genera with potential functional relevance, we further compared the taxonomic composition of gut microbiota between healthy individuals and patients with inflammatory bowel disease. At the genus level (Figure 2A), multiple pro-inflammatory genera were significantly enriched in IBD patients, including *g_Fusobacterium* (*p* < 0.001), *g_Morganella* (*p* < 0.001), *g_Anaeroglobus* (*p* < 0.001), and *g_Veillonella* (*p* < 0.05). These genera, known as pathogens or opportunistic bacteria, can exacerbate intestinal inflammation and disrupt mucosal barrier integrity through multiple mechanisms, thereby collectively contributing to the progression of IBD (Anani et al., 2019; Khorsand et al., 2022; Wang et al., 2024).

In sharp contrast, the gut microbiota of healthy individuals was characterized by a predominance of SCFAs producing commensals. Among them, *g_Agathobacter* (*p* < 0.0001) and *g_Coprococcus* (*p* < 0.0001) were major butyrate producers that provide energy to intestinal epithelial cells and sustain an anti-inflammatory environment (Singh et al., 2022; Visuthranukul et al., 2024). *g_Alistipes* (*p* < 0.0001) participated in bile acid metabolism and immune modulation (David et al., 2014), whereas multiple *g_Ruminococcus* subgroups including *g_Ruminococcus_C* (*p* < 0.0001), *g_Ruminococcus_D* (*p* < 0.0001), and *g_Ruminococcus_E* (*p* < 0.0001) were primarily responsible for degrading complex dietary fibers (Arnold et al., 2021). In addition, *g_Hungatella_A* (*p* < 0.0001) and *g_Acetatifactor* (*p* < 0.0001) contributed to beneficial microbial metabolic activities (Jiao et al., 2024; Pathak et al., 2018). Such a commensal-dominated ecological configuration forms the fundamental basis for maintaining intestinal homeostasis and suppressing excessive immune activation. The pronounced depletion of these protective taxa in IBD patients directly indicates a severe impairment of gut microbial defense functions.

We further performed receiver operating characteristic (ROC) analysis at the species level to evaluate the diagnostic potential of taxa exhibiting significant differences between groups (Figure 2B). Several species showed strong discriminatory power between healthy individuals and IBD patients. Beneficial taxa enriched in healthy controls included *s_Hungatella_A_sp003478355* (AUC: 0.859), *s_Acetatifactor_sp900066365* (AUC: 0.873), *s_Agathobacter faecis* (AUC: 0.814), *s_Coprococcus eutactus_A* (AUC: 0.793), *s_Alistipes onderdonkii* (AUC: 0.828), *s_Ruminococcus_C_sp000980705* (AUC: 0.857), *s_Ruminococcus_D_bicirculans* (AUC: 0.809), and *s_Ruminococcus_E_bromii* (AUC: 0.824). The reduced abundance of these taxa may be closely associated with IBD pathogenesis. Conversely, several potentially pathogenic species were enriched in IBD patients, including *s_Fusobacterium periodonticum_D* (AUC: 0.677), *s_Morganella morganii_B* (AUC: 0.713), and *s_Anaeroglobus geminatus* (AUC: 0.698). Notably, *s_Veillonella parvula* exhibited relatively limited discriminative ability (AUC: 0.663).

These findings suggest that the concurrent depletion of SCFA-producing commensals and enrichment of potential pathogens defines the microbial signature of IBD, and several species identified here hold promise as noninvasive fecal diagnostic biomarkers. This pattern of dysbiosis may underlie the disruption of intestinal metabolic and immune homeostasis, providing potential targets for microbiota-based diagnostics and interventions.

### Microbial metabolic dysfunction in IBD patients

**Figure 3:**
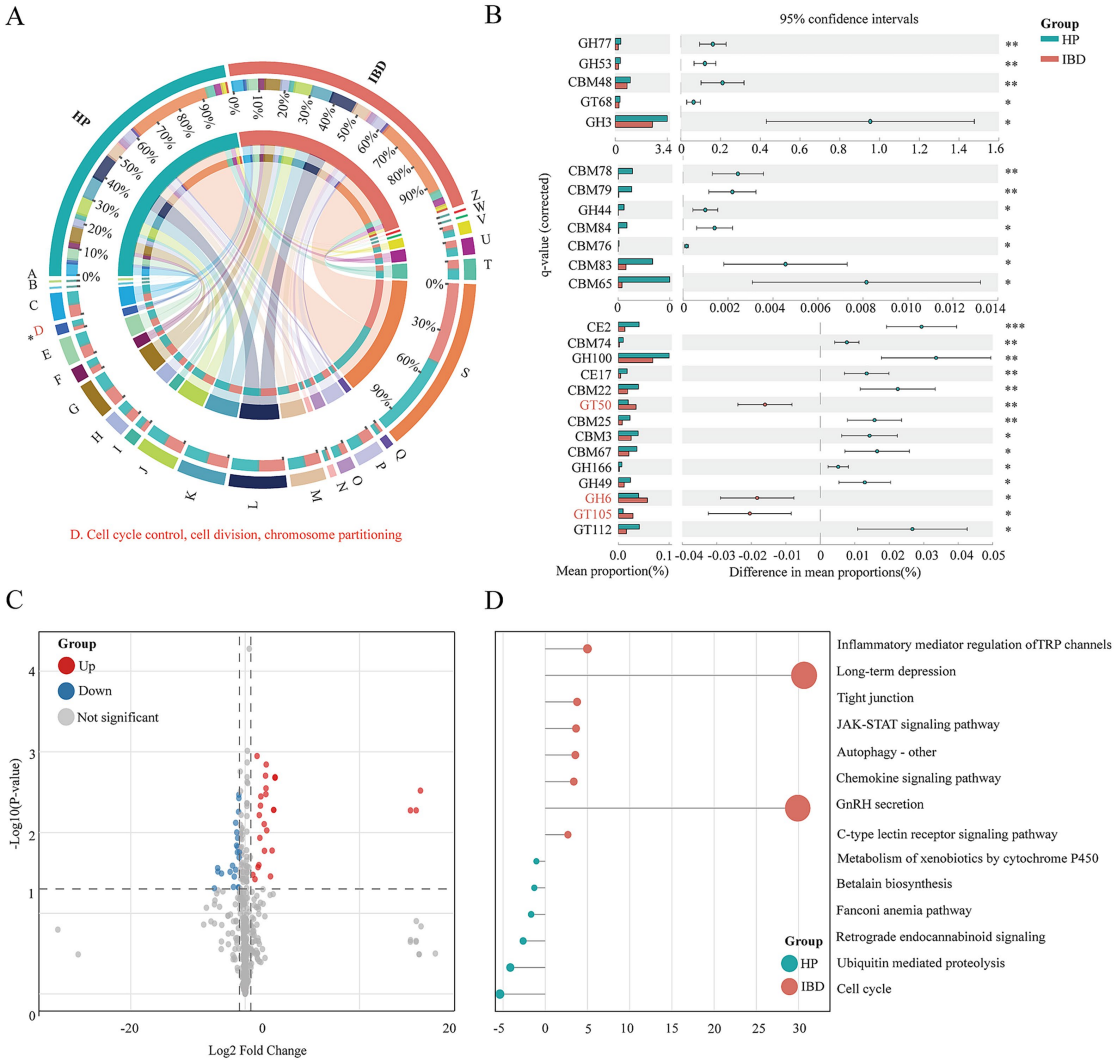
Functional composition analysis of the microbial community between healthy individuals (HP, *n* = 47) and patients with IBD (*n* = 40). **(A)** Chord diagram of clusters of orthologous groups (COG) functional categories, illustrating the distribution and associations of protein functions. **(B)** STAMP bar plot of CAZyme (carbohydrate-active enzymes) profiles, showing differential abundances of enzyme families between groups; the statistically significant marker ∗ represents * for *p* ≤ 0.05; ** for *p* ≤ 0.01; and *** for *p* ≤ 0.001. **(C)** Volcano plot of KEGG pathway enrichment. Red dots represent pathways significantly upregulated in the IBD group, blue dots represent pathways downregulated in IBD, and gray dots indicate pathways with no significant difference. **(D)** Lollipop chart of significantly different KEGG pathways, displaying the effect size and statistical significance of each pathway.

To delineate the functional characteristics of the gut microbiota, we performed COG, CAZyme, and KEGG based annotations of the predicted genes. COG analysis (Figure 3A) revealed a significant difference between healthy controls and IBD patients in only one functional Category cell cycle control, cell division, and chromosome partitioning (Category D, Supplementary Figure S2) suggesting potential disturbances in bacterial proliferation and genomic stability within the IBD associated microbiota.

Further analysis based on the CAZyme database revealed pronounced alterations in carbohydrate metabolism between the two groups (Figure 3B). The gut microbiota of healthy individuals was significantly enriched in a broad range of carbohydrate active enzymes, including glycoside hydrolases (GHs) and carbohydrate-binding modules (CBMs) involved in starch and glycogen degradation, such as GH77 (*p* < 0.01), GH53 (*p* < 0.01), and CBM48 (*p* < 0.01), as well as hemicellulose and pectin degrading enzymes including GH3 (*p* < 0.05) and GH44 (*p* < 0.05). Additional enrichment of GH100 (*p* < 0.01) and CE17 (*p* < 0.01) further indicated the strong capacity of healthy microbiota to decompose complex dietary fibers and polysaccharides, thereby sustaining SCFAs production and microbial ecological stability. In contrast, the IBD microbiota displayed a distinct functional profile, with significant enrichment of GT50 (*p* < 0.01), GH6 (*p* < 0.05), and GT105 (*p* < 0.05). This functional shift was characterized by a general reduction in fiber-degrading potential and a relative enhancement of bacterial cell wall biosynthesis and cellulose-specific degradation pathways, reflecting possible adaptive remodeling of microbial metabolism in response to the inflammatory gut environment associated with IBD.

We next conducted a systematic comparison of metabolic pathways between the two groups. In total, 45 KEGG pathways were significantly different, with 25 upregulated in the IBD group and 20 enriched in healthy controls (Supplementary Table S2). At the KEGG level 2 classification, eight differential categories were identified (Supplementary Figure S3), healthy controls exhibited higher activity in growth hormone synthesis, carbohydrate metabolism, proteasome, and cell growth, whereas the IBD group showed significant enrichment in amino acid metabolism, secondary metabolism, cofactor metabolism, and proteoglycans in cancer.

At the more refined KEGG level 3 (Figure 3C), the IBD group exhibited enrichment of multiple pathways related to immune and inflammatory regulation, xenobiotic metabolism, and cell signaling (Figure 3D), including inflammatory mediator regulation of TRP channels [ko04750], chemokine signaling pathway [ko04062], c-type lectin receptor signaling pathway [ko04625], and JAK–STAT signaling pathway [ko04630]. In addition, pathways such as autophagy – other [ko04136], tight junction [ko04530], and long-term depression [ko04730] were also more active in IBD patients. In contrast, healthy controls were enriched in pathways associated with cellular homeostasis and detoxification, including metabolism of xenobiotics by cytochrome P450 [ko00980], betalain biosynthesis [ko00965], fanconi anemia pathway [ko03460], retrograde endocannabinoid signaling [ko04723], ubiquitin-mediated proteolysis [ko04120], and cell cycle [ko04110].

Collectively, these functional and metabolic differences indicate that the gut microbiota in IBD patients may promote intestinal inflammation and disrupt homeostasis by activating immune and inflammatory signaling, enhancing xenobiotic metabolism, and altering cell signaling pathways, while simultaneously weakening microbial and host mechanisms that maintain cellular stability.

### Interaction networks and functional associations of gut microbiota in healthy individuals and IBD patients

**Figure 4:**
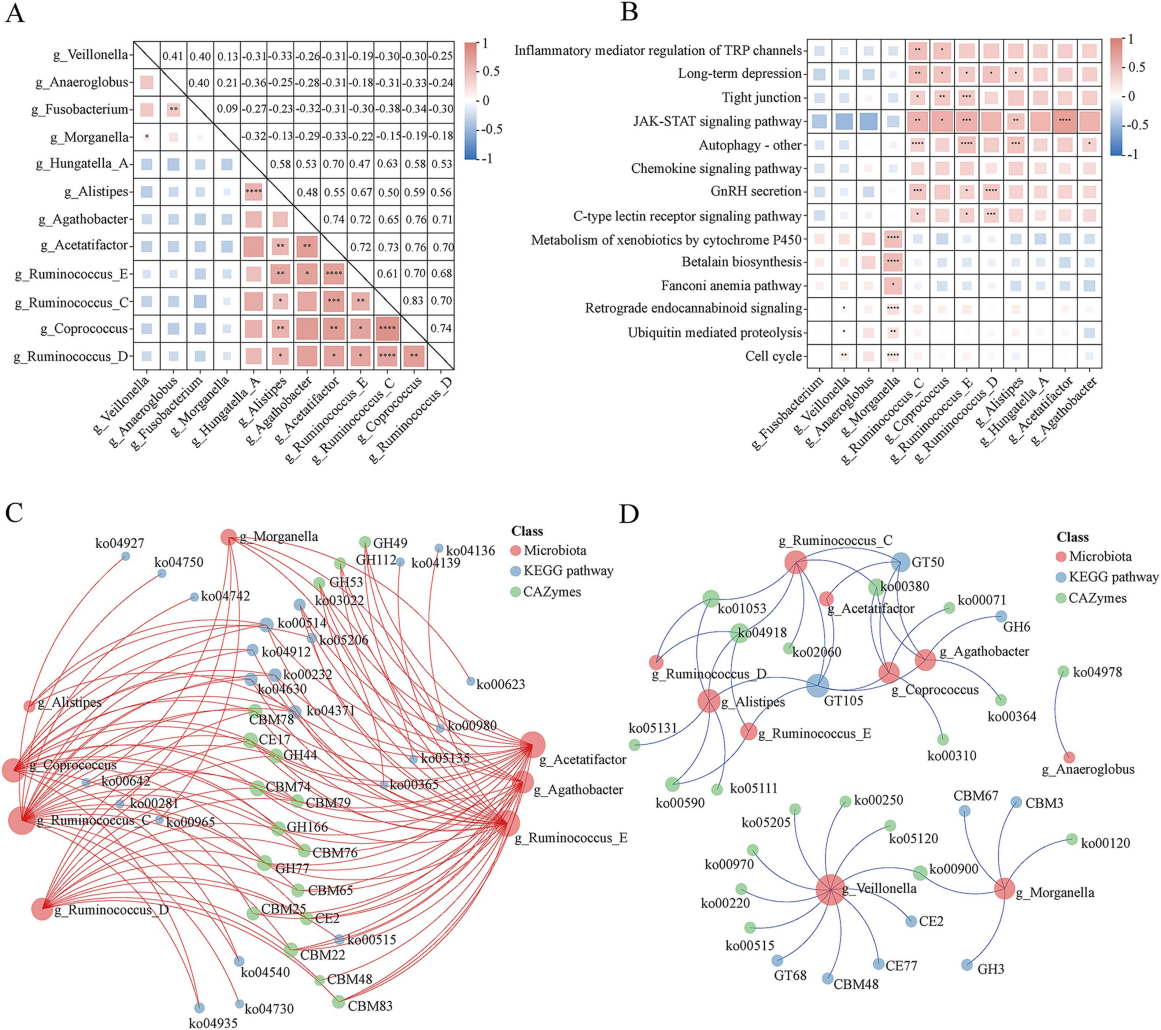
Integrated correlation analysis of microbial and functional profiles between healthy individuals (HP, *n* = 47) and patients with IBD (*n* = 40). **(A)** Spearman correlation matrix among differentially abundant genera. Red and blue colors represent significant positive and negative correlations, respectively; the statistically significant marker ∗ represents * for *p* ≤ 0.05; ** for *p* ≤ 0.01; *** for *p* ≤ 0.001; and **** for *p* ≤ 0.0001. **(B)** Spearman correlation analysis between microbial genera and significantly different KEGG level-3 pathways. Red indicates a positive correlation, while blue indicates a negative correlation; the statistically significant marker ∗ represents * for *p* ≤ 0.05; ** for *p* ≤ 0.01; *** for *p* ≤ 0.001; and **** for *p* ≤ 0.0001. **(C)** Co-occurrence network illustrating significant positive correlations (*R* > 0.5) among differentially abundant genera, KEGG pathways, and CAZyme families. **(D)** Co-occurrence network of significant negative correlations (*R* > 0.5) among the differentially abundant genera, KEGG pathways, and CAZyme families.

To further elucidate the interaction characteristics of gut microbial communities in healthy individuals and IBD patients, we performed a Spearman correlation analysis. The genus-level correlation heatmap revealed distinct clustering patterns between the two groups (Figure 4A). In the IBD group, four pro-inflammatory *g_Veillonella*, *g_Anaeroglobus*, *g_Fusobacterium*, and *g_Morganella* were significantly enriched and showed strong positive correlations with each other, with the strongest association observed between *g_Anaeroglobus* and *g_Fusobacterium* (*r* = 0.40). In contrast, the eight genera enriched in healthy individuals *g_Hungatella_A*, *g_Alistipes*, *g_Agathobacter*, *g_Acetatifactor*, *g_Ruminococcus_E*, *g_Ruminococcus_C*, *g_Coprococcus*, and *g_Ruminococcus_D* formed a tightly coordinated positive correlation cluster, with the highest correlation observed between *g_Coprococcus* and *g_Ruminococcus_C* (*r* = 0.83). Notably, genera associated with IBD and those associated with healthy controls were generally negatively correlated, among which *g_Ruminococcus_C* and *g_Fusobacterium* showed the strongest negative correlation (*r* = −0.38).

To further integrate microbial and functional features, we correlated differential genera with KEGG level 3 pathways (Figure 4B). The IBD-enriched genus *g_Morganella* showed strong positive correlations with several pathways that were more active in healthy individuals, including metabolism of xenobiotics by cytochrome P450 (ko00980), betalain biosynthesis (ko00965), fanconi anemia pathway (ko03460), retrograde endocannabinoid signaling (ko04723), ubiquitin-mediated proteolysis (ko04120), and cell cycle (ko04110). Interestingly, although the inflammatory pathway inflammatory mediator regulation of TRP channels (ko04750) was globally enriched in the IBD group, its activity was positively correlated with the abundances of the health associated genera *g_Coprococcus* and *g_Ruminococcus_C*. Moreover, *g_Ruminococcus_C*, *g_Ruminococcus_E*, and *g_Alistipes* were significantly positively correlated with the autophagy – other (ko04136) pathway.

To systematically map the global associations between microbes and functional modules, we constructed an integrated correlation network encompassing genera, KEGG pathways, and CAZyme families. In the positive correlation network (Figure 4C), *g_Ruminococcus_C*, *g_Ruminococcus_E*, and *g_Acetatifactor* emerged as key functional hubs with the highest connectivity, suggesting their central ecological roles. Specifically, *g_Ruminococcus_C* exhibited the most extensive functional associations, showing positive correlations not only with multiple carbohydrate-active enzymes but also with the other glycan degradation pathway (ko00514), highlighting its potential as a key fiber-degrading taxon. In addition, *g_Ruminococcus_C* was positively correlated with several host-related signaling pathways, including endocrine regulatory pathways such as growth hormone synthesis and secretion (ko04935), prolactin signaling (ko04927), and GnRH secretion (ko04912), immune and signal transduction pathways such as JAK–STAT signaling (ko04630), apolipoprotein-E signaling (ko04371), and inflammatory mediator regulation of TRP channels (ko04750); and neural function pathways such as long-term depression (ko04730) and taste transduction (ko04742), suggesting a potential involvement of this genus in host physiological regulation via the gut–brain axis. Similarly, *g_Ruminococcus_E* and *g_Acetatifactor* were positively correlated with multiple polysaccharide degradation functions and several CBM modules, as well as host-associated pathways including JAK–STAT signaling (ko04630), apolipoprotein-E signaling (ko04371), and caffeine metabolism (ko00232). Notably, *g_Ruminococcus_E* was also positively correlated with proteasome (ko03022), autophagy (ko04136), and mitophagy (ko04139), implying potential roles in protein homeostasis and cellular maintenance.

In the negative correlation network (Figure 4D), a distinct interaction pattern was observed. *g_Alistipes* showed negative correlations with arachidonic acid metabolism (ko00590), thyroid hormone synthesis (ko04918), and several pathogen infection pathways, including shigellosis (ko05131) and *vibrio cholerae* infection (ko05111). *g_Morganella* was negatively correlated with primary bile acid biosynthesis (ko00120) and terpenoid backbone biosynthesis (ko00900), while *g_Veillonella* exhibited broad negative correlations with amino acid and nucleotide metabolism pathways, including aminoacyl-tRNA biosynthesis (ko00970), adherens junction (ko05120), and arginine biosynthesis (ko00220). Interestingly, the core genus *g_Ruminococcus_C* also appeared in the negative network, showing negative correlations with thyroid hormone synthesis (ko04918) and the phosphotransferase system (PTS) (ko02060).

Collectively, these multi-layered correlation analyses identified *g_Ruminococcus_C*, *g_Ruminococcus_E*, and *g_Acetatifactor* as central symbiotic genera with broad functional associations and cooperative interactions, while *g_Alistipes*, *g_Morganella*, and *g_Veillonella* displayed negative correlations with specific host metabolic and immune pathways. These findings provide new insights into the potential ecological differentiation and functional specialization of gut microbes in the context of IBD pathogenesis.

### Response patterns and contribution of environmental factors to gut microbial composition

**Figure 5:**
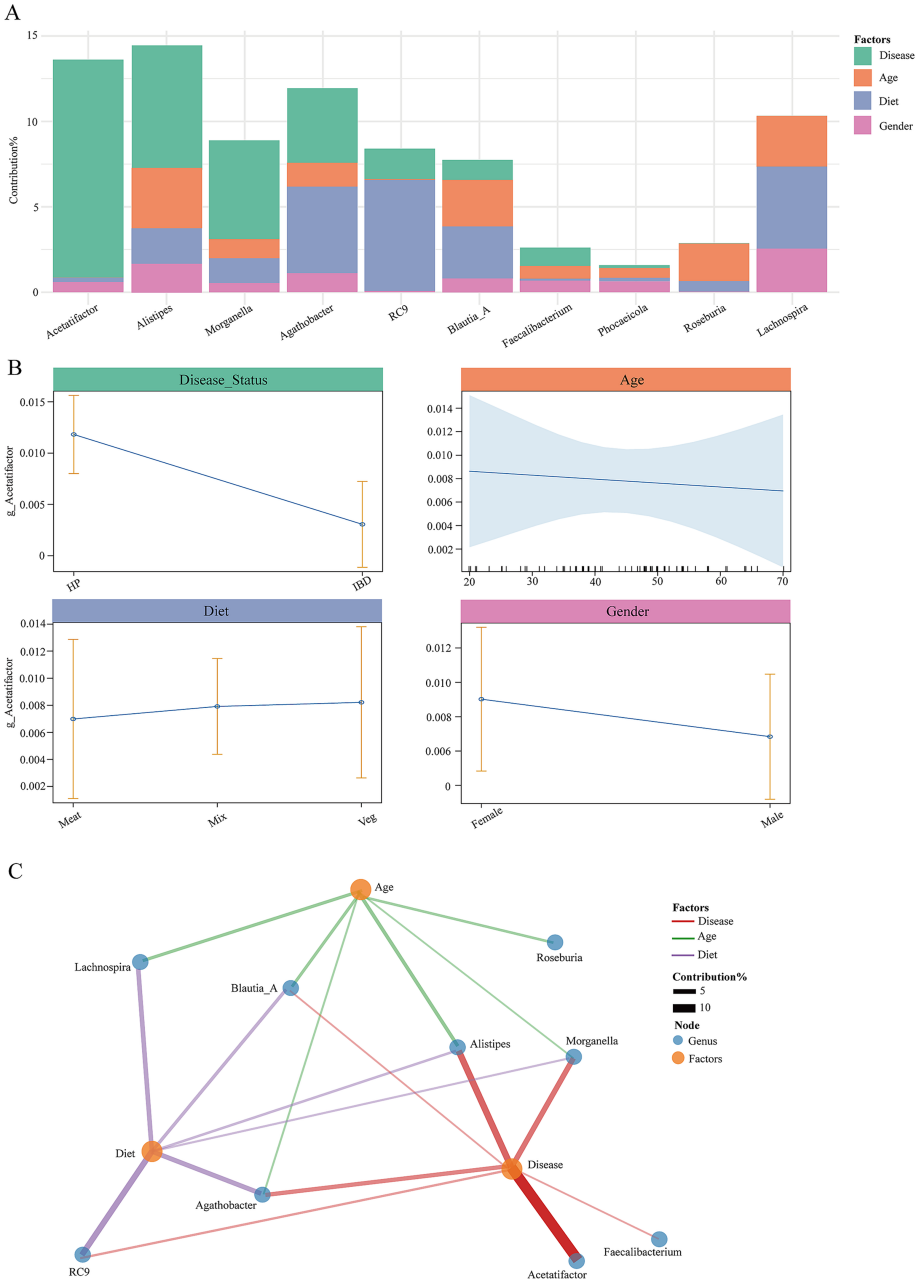
Environmental factors shaping gut microbial composition. **(A)** Variance explained (*R*^2^) by different factors for key genera. Different colored bars represent different environmental factors. **(B)** Model effect plots showing genus abundance changes across disease states, age, and diet. **(C)** Interaction network between factors (yellow nodes) and genera (blue nodes). Edge thickness represents the strength of the association.

Using a linear mixed-effects model, we quantified the relative contributions of environmental factors to gut microbial variation (Figure 5A). The explained variance (*R*^2^) ranged from 1.60 to 14.57%, with the highest value observed for *g_Alistipes* (14.57%) and the lowest for *g_Phocaeicola* (1.60%). Among all tested factors, disease status emerged as the dominant determinant of community composition, showing a particularly strong effect on *g_Acetatifactor* (12.75%), which was minimally influenced by other variables suggesting its potential as a disease-specific biomarker.

In contrast, *g_Alistipes* exhibited a multi-factor responsive pattern, being significantly associated with disease status (7.28%), age (3.53%), and diet type (2.08%). Diet also exerted marked effects on *g_RC9* (6.51%) and *g_Lachnospira* (4.81%), while sex contributed modestly, with the largest effect observed for *g_Lachnospira* (2.54%). Collectively, these results indicate distinct environmental response modes, *g_Acetatifactor* showed a disease-specific response, *g_RC9* was diet-driven, and *g_Alistipes* and *g_Blautia_A* responded to multiple environmental cues. Model effect plots confirmed these associations (Figure 5B), *g_Acetatifactor* abundance varied significantly across disease states, *g_Alistipes* and *g_RC9* showed gradual abundance shifts with age and diet, and *g_Lachnospira* was jointly influenced by diet and sex (Supplementary Figure S4).

Network analysis further revealed a complex interaction architecture among taxa and environmental factors (Figure 5C). The network comprised nine nodes and 19 edges, with disease status connected to seven genera highlighting its central role in community assembly. Both age and diet were linked to six genera, forming distinct interaction modules. Hub analysis identified *g_Blautia_A*, *g__Alistipes*, and *g_Morganella* as key taxa associated with multiple environmental variables, suggesting their pivotal ecological roles in mediating microbial responses to host and environmental perturbations. Together, these findings demonstrate that gut microbial composition is shaped by a hierarchical interplay of disease, diet, and host-related factors, with certain genera acting as ecological sensors and functional stabilizers within the intestinal ecosystem.

In summary, our results highlight a hierarchical regulatory framework in which disease status exerts the strongest selective pressure on microbial community structure, whereas dietary and host-related variables contribute to fine-scale ecological modulation and adaptive functional responses.

## Discussion

**Figure 6:**
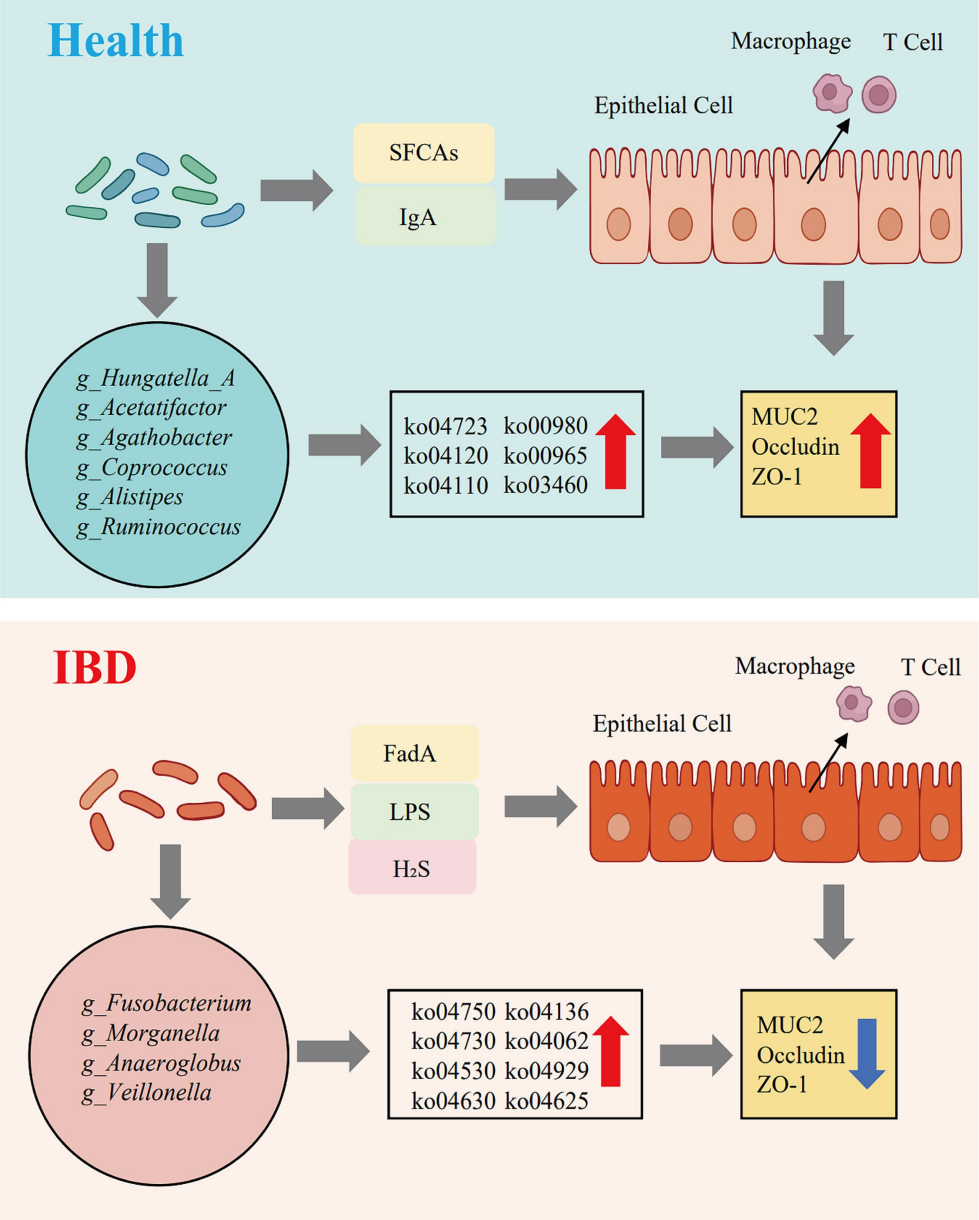
Dysbiotic evolution of the gut microbiome and potential pro-inflammatory mechanisms in the progression from health to inflammatory bowel disease.

Inflammatory bowel disease (IBD) is characterized by chronic intestinal inflammation accompanied by marked disruption of the gut microbial ecosystem. To gain a comprehensive view of these ecological perturbations, we conducted metagenomic sequencing of fecal samples from 47 healthy individuals and 40 patients with IBD. The integrative analysis uncovered profound alterations in the gut microbiome across three interconnected dimensions community composition, functional potential, and environmental responsiveness (Figure 6).

### Expansion of pathogenic taxa and depletion of beneficial commensals: a structural imbalance of the IBD-associated gut microbiota

We further revealed a profound dysbiosis of the gut microbiota in IBD patients, characterized not merely by alterations in microbial abundance but by a structural reorganization featuring the expansion of pathobionts and depletion of commensals. Such ecological disruption not only perpetuates local intestinal inflammation but may also act as a driving force for tumorigenesis at distal sites.

Among the pro-inflammatory taxa, the enrichment of *g_Veillonella* and *g_Fusobacterium* emerged as a hallmark of the IBD-associated microbiome. Gram-negative bacteria within the genus *g_Veillonella* produce a distinctive lipooligosaccharide-type LPS with tetra-acylated lipid A, a weak TLR4 agonist that nonetheless triggers chronic, low-grade immune activation during dysbiosis (Shan et al., 2022). This persistent stimulation promotes a pro-tumorigenic microenvironment through constitutive activation of the NF-κB–IL-6–STAT3 signaling axis, which is a well-documented driver of inflammation-associated carcinogenesis (Park et al., 2009; Zhan et al., 2022). *g_Anaeroglobus*, a close relative of *g_Veillonella*, likely shares this immunomodulatory property, suggesting a similar contribution to chronic inflammatory activation (Carlier et al., 2002).

Meanwhile, *g_Fusobacterium* is recognized as a critical initiator of colorectal inflammation and cancer. Its adhesin FadA enables epithelial invasion and NF-κB activation, leading to excessive cytokine release and barrier disruption (Pei et al., 2025). In parallel, the expansion of *g_Morganella* may further amplify intestinal inflammation via secretion of the pore-forming toxin HpmA, which damages epithelial membranes, particularly those of goblet cells, and induces the release of TNF-α, IL-1β, and IL-6, thereby forming a self-reinforcing inflammatory loop (Liu et al., 2016). Notably, our correlation network analysis revealed that *g_Morganella*, *g_Fusobacterium*, and *g_Anaeroglobus* formed a highly interconnected positive cluster in IBD samples, suggesting that these taxa constitute a cooperative pro-inflammatory microbial network that perpetuates chronic intestinal inflammation.

In healthy individuals, the gut microbiota is dominated by fiber-degrading commensals such as *g_Ruminococcus* (including *g_Ruminococcus_C*, *g_Ruminococcus_D*, and *g_Ruminococcus_E*), *g_Agathobacter*, and *g_Coprococcus*, which are profoundly depleted in IBD. These taxa form the metabolic backbone for SCFA production, sustaining epithelial energy metabolism and mucosal immune homeostasis (Dicks, 2024; Pan et al., 2024; Rodríguez-García et al., 2024). Among them, butyrate plays a pivotal anti-inflammatory role by preserving barrier integrity and inhibiting M1 macrophage polarization (Cai et al., 2025). The loss of these SCFA-producing bacteria disrupts the protective metabolic network and undermines immune tolerance, establishing a dysbiotic state defined by commensal depletion and pathobiont expansion, which represents a central ecological and pathogenic signature of IBD.

Several previous metagenomic studies have reported patterns of gut microbiota alterations in IBD that are broadly consistent with our findings. For example, patients with IBD typically exhibit reduced microbial diversity and diminished abundance of SCFA-producing taxa such as *Ruminococcus*, *Faecalibacterium*, *Roseburia*, and other Firmicutes, which are involved in carbohydrate fermentation and mucosal immune regulation. These changes are associated with lower levels of SCFAs and disrupted intestinal homeostasis in IBD compared with healthy controls (Lo Sasso et al., 2021). However, differences between studies can arise due to geographic, dietary, and population-specific factors that shape the baseline gut microbiome of healthy individuals. Reference metagenomic analyses of healthy cohorts indicate that the gut microbiota varies substantially across populations, with core functions such as carbohydrate metabolism and SCFA production being broadly conserved but taxonomic profiles showing variability related to lifestyle, diet, and environmental exposures. Such variation may partly explain why specific taxa enriched or depleted in our cohort differ from those reported in other regions or study populations (Rinninella et al., 2019).

### Functional remodeling of the gut microbiome: a shift from nutrient metabolism to inflammatory adaptation

Comprehensive metagenomic annotation revealed changes in functional characteristics of the gut microbiome in patients with inflammatory bowel disease. CAZyme-based profiling showed that healthy microbiota harbor a highly efficient and diverse capacity for complex polysaccharide degradation, consistent with their enrichment in SCFAs producing taxa that form the metabolic core for energy harvesting and host nourishment. In contrast, this function was markedly impaired in the IBD microbiome, where enriched CAZyme families were predominantly associated with bacterial cell wall biosynthesis (Lan et al., 2023). This shift suggests that under inflammatory stress, microbial communities undergo a strategic transition from mutualistic nutrient exchange toward self-preservation and survival.

KEGG pathway enrichment further supported this notion by revealing that the C-type lectin receptor signaling pathway was significantly enriched in IBD-associated microbiota, suggesting that dysbiotic microbes may act as key triggers of innate immune activation. Such activation could directly couple to central inflammatory transducers. Consistent with findings in *Bombyx mori*, where C-type lectin 5 functions as a pattern-recognition receptor that directly activates the JAK–STAT pathway (Geng et al., 2021). Moreover, microbiota-derived metabolites such as SCFAs, tryptophan derivatives, and secondary bile acids regulate host immune pathways via receptors including AhR, FXR, and TGR5, and their dysregulation in IBD has been associated with enhanced NF-κB activation and intestinal inflammation (Hu et al., 2022). Our results suggest the existence of an evolutionarily conserved mechanism in which specific microbial components engage C-type lectin receptors to initiate JAK–STAT signaling in the human gut. This provides a new molecular framework for understanding how the microbiota may bypass conventional cytokine cascades to rapidly and directly drive chronic inflammation. Microbiota-dependent tryptophan metabolites can engage AhR and PXR pathways, modulating intestinal immune responses and contributing to the balance between pro- and anti-inflammatory signaling in the gut (Pernomian et al., 2020). Once activated, the JAK–STAT signaling pathway functions as a central amplifier of inflammatory responses. As a convergent downstream node of multiple pro-inflammatory cytokines, JAK–STAT signaling has been firmly established as a pivotal axis in autoimmune diseases (Xue et al., 2023). Our data extend this paradigm to the microbial etiology of IBD, suggesting that dysbiotic communities not only contribute through their own structural components but also by shaping a cytokine-enriched microenvironment that sustains persistent JAK–STAT activation. This chronic signaling loop may contribute to the observed inflammation and altered immune cell differentiation in IBD, although causal relationships remain to be verified. Furthermore, enrichment of the inflammatory mediator regulation of TRP channels pathway provides a mechanistic link to the visceral pain and neurogenic inflammation frequently observed in IBD (Raskov et al., 2016). TRP channels, particularly TRPV1 and TRPA1 act as key molecular sensors bridging tissue injury and neuronal excitation (Liang et al., 2023). These channels can be sensitized by diverse inflammatory mediators, including bradykinins and prostaglandins, thereby triggering pain perception and neurogenic inflammation (Gouin et al., 2017). Such activation promotes vasodilation, plasma extravasation, and immune cell infiltration. Within the inflamed intestinal milieu, a dysbiosis-driven cytokine environment may chronically sensitize enteric sensory neurons via this pathway, contributing not only to abdominal pain but also to the amplification of mucosal immune injury through neuroimmune crosstalk (Sun et al., 2025).

The microbiota of healthy individuals showed enrichment in pathways such as Ubiquitin-mediated proteolysis [ko04120] and the Cell cycle [ko04110], which are essential for maintaining host cellular homeostasis and detoxification. Previous studies have demonstrated that the ubiquitin-mediated proteolysis pathway facilitates the clearance of aberrant proteins generated under metabolic or oxidative stress, thereby preserving normal cellular function and preventing stress-induced cytotoxic aggregation and cell death (Zheng and Shabek, 2017). Meanwhile, the cell cycle pathway supports the normal proliferation and renewal of intestinal epithelial cells (Glaviano et al., 2025). Collectively, these findings suggest that microbial functional dysregulation in IBD reflects a coexistence of diminished protective metabolic capacity and enhanced pro-inflammatory signaling potential, jointly shaping a persistently inflamed and self-sustaining intestinal microenvironment.

### Environmental shaping of the gut microbiota and genus-specific response patterns

The composition of the gut microbiota arises from complex interactions among host genetics, immune status, and environmental factors. Using a linear mixed-effects model, we quantified the relative contribution of non-genetic environmental variables to variations in key bacterial genera. The results revealed distinct response patterns across taxa, consistent with previous observations that environmental factors can profoundly reshape microbial communities (Donald and Finlay, 2023). For example, the variation of genera such as *g_Acetatifactor* was almost entirely determined by disease status, showing minimal sensitivity to other environmental parameters. This finding suggests that *g_Acetatifactor* may serve as a highly specific biomarker reflecting intestinal inflammation in the host (Umemura et al., 2024). In contrast, taxa represented by g_RC9 were primarily driven by dietary structure, highlighting the potential of nutritional modulation as an effective approach for microbiome-based intervention (Schneider et al., 2024). Notably, genera such as *g_Alistipes* exhibited integrated responses to multiple factors including disease state, age, and diet, suggesting their role as ecological sensors that integrate environmental cues within the gut ecosystem (Messaoudene et al., 2022). These taxon-specific response patterns reflect significant differences in ecological niche breadth and adaptive flexibility among microbial lineages. Overall, the remodeling of the IBD-associated microbiota appears to result from the combined influences of disease state and environmental factors, which together drive the characteristic dysbiosis observed in IBD. These findings offer new perspectives for precision microbiota-based therapies.

We have several limitations. First, the cross-sectional design precludes inferences about temporal dynamics or causal relationships between gut microbiota alterations and IBD progression. Second, detailed data on medication history and dietary habits were not systematically collected for all participants; these unmeasured confounding factors may have influenced the observed differences in microbial composition. Additionally, the sample size was relatively limited, and all participants were recruited from a single geographic region, which may affect the generalizability of the findings and the statistical power for subgroup analyses. Future studies should consider implementing large-scale, multicenter longitudinal cohorts to clarify the causal links between microbial dynamics and disease course. Stratified analyses based on IBD subtypes (e.g., CD vs. UC) are needed to identify subtype-specific microbial signatures. Furthermore, animal models or *in vitro* experiments could help validate the molecular mechanisms underlying the interactions between key microbial taxa and the host.

## Conclusion

We conducted a comprehensive metagenomic analysis to characterize gut microbiome dysbiosis in IBD. Our results reveal that beneficial metabolic networks centered on dietary fiber degradation and short-chain fatty acid production are associated with a relative decrease, while pro-inflammatory bacterial consortia appear to be enriched. Moreover, the overall microbial metabolic appears to shift from mutualistic cooperation with the host toward adaptation to an inflammatory environment. Collectively, these findings indicate that coordinated alterations in microbial composition and function, together with the dominant influence of disease status, may contribute to a pro-inflammatory microenvironment and provide new insight into potential microbial contributions to the pathophysiology of IBD.

## Data Availability

The raw metagenomic sequencing data have been deposited in the NCBI Sequence Read Archive (SRA) under BioProject accession PRJCA038885. The project is also accessible via the CNCB-NGDC repository at: https://ngdc.cncb.ac.cn/gsub/submit/bioproject/PRJCA038885/overview.
