# Supplementary material for: Disease-driven restructuring of the gut microbiome underlies inflammatory bowel disease dysbiosis
Source: Front Microbiol. 2026 Jan 27;16:1744574. doi: 10.3389/fmicb.2025.1744574 (PMC12888223; doi:10.3389/fmicb.2025.1744574)
Supplement: Supplementary file 1 [file Supplementary_file_1.docx]

| **Supplement Table S1. Baseline characteristics of the study participants.** | | | | |
| --- | --- | --- | --- | --- |
| **ID** | **Group** | **Gender** | **Age** | **Diet** |
| 1003 | HP | Female | 39 | Mix |
| 1004 | HP | Male | 51 | Mix |
| 1006 | HP | Female | 54 | Mix |
| 1007 | HP | Male | 49 | Meat |
| 1008 | HP | Female | 51 | Mix |
| 1010 | HP | Female | 48 | Mix |
| 1011 | HP | Male | 40 | Mix |
| 1012 | HP | Female | 41 | Veg |
| 1016 | HP | Female | 56 | Veg |
| 1018 | HP | Female | 48 | Veg |
| 1019 | HP | Male | 47 | Meat |
| 1020 | HP | Female | 65 | Mix |
| 1021 | HP | Female | 58 | Mix |
| 1025 | HP | Male | 51 | Meat |
| 1026 | HP | Female | 50 | Meat |
| 1035 | HP | Female | 66 | Veg |
| 1039 | HP | Male | 55 | Veg |
| 1040 | HP | Female | 66 | Mix |
| 1041 | HP | Female | 38 | Veg |
| 1043 | HP | Male | 35 | Meat |
| 1044 | HP | Male | 61 | Mix |
| 1048 | HP | Male | 36 | Mix |
| 1049 | HP | Female | 48 | Veg |
| 1054 | HP | Female | 37 | Mix |
| 1055 | HP | Male | 53 | Meat |
| 1060 | HP | Female | 50 | Veg |
| 1061 | HP | Female | 39 | Mix |
| 1063 | HP | Female | 34 | Mix |
| 1067 | HP | Female | 44 | Mix |
| 1068 | HP | Male | 48 | Veg |
| 1070 | HP | Male | 51 | Mix |
| 1071 | HP | Female | 64 | Mix |
| 1072 | HP | Female | 70 | Mix |
| 1073 | HP | Male | 52 | Mix |
| 1074 | HP | Male | 54 | Veg |
| 1081 | HP | Female | 54 | Mix |
| 1082 | HP | Female | 61 | Mix |
| 1005 | HP | Female | 64 | Mix |
| 1030 | HP | Female | 64 | Veg |
| 1031 | HP | Female | 54 | Veg |
| 1032 | HP | Female | 49 | Mix |
| 1036 | HP | Male | 50 | Veg |
| 1042 | HP | Male | 48 | Meat |
| 1046 | HP | Male | 54 | Mix |
| 1052 | HP | Male | 46 | Mix |
| 1056 | HP | Male | 35 | Mix |
| 1057 | HP | Male | 71 | Mix |
| 2002 | UC | Male | 20 | Veg |
| 2006 | CD | Male | 29 | Mix |
| 2009 | CD | Male | 18 | Mix |
| 2010 | CD | Male | 35 | Veg |
| 2012 | CD | Male | 28 | Meat |
| 2014 | UC | Male | 49 | Mix |
| 2017 | CD | Male | 74 | Meat |
| 2019 | CD | Male | 39 | Veg |
| 2021 | UC | Female | 29 | Mix |
| 2022 | CD | Male | 31 | Meat |
| 2023 | CD | Male | 20 | Mix |
| 2024 | UC | Female | 58 | Mix |
| 2025 | CD | Male | 18 | Mix |
| 2027 | CD | Male | 26 | Mix |
| 2029 | CD | Female | 16 | Mix |
| 2030 | UC | Male | 41 | Mix |
| 2032 | CD | Male | 48 | Mix |
| 2033 | CD | Male | 37 | Meat |
| 2034 | CD | Female | 21 | Mix |
| 2035 | CD | Male | 23 | Mix |
| 2036 | UC | Male | 58 | Mix |
| 2037 | UC | Male | 48 | Meat |
| 2038 | CD | Male | 25 | Meat |
| 2040 | CD | Female | 53 | Meat |
| 2042 | UC | Female | 53 | Mix |
| 2043 | CD | Female | 47 | Veg |
| 2044 | UC | Female | 40 | Mix |
| 2045 | CD | Male | 48 | Meat |
| 2047 | CD | Male | 32 | Meat |
| 2048 | UC | Male | 40 | Mix |
| 2053 | UC | Male | 21 | Mix |
| 2056 | CD | Male | 45 | Mix |
| 2057 | CD | Male | 37 | Meat |
| 2058 | CD | Male | 38 | Mix |
| 2059 | UC | Female | 54 | Mix |
| 2060 | CD | Male | 51 | Mix |
| 2061 | CD | Female | 52 | Veg |
| 2062 | UC | Female | 65 | Veg |
| 2063 | CD | Male | 25 | Veg |
| 2064 | CD | Male | 25 | Veg |

**Note:** HP stands for healthy person, CD for Crohn's disease, and UC for ulcerative colitis.

**Supplement Table S2. Significantly altered KEGG pathways at level 3.**

| **Pathway** | **Log2FC** | **P_value** | **negLog10P** | **Significant** |
| --- | --- | --- | --- | --- |
| Secretion and action [PATH:ko04935] | 5.23 | 0.01 | 2.68 | Up regulated |
| Amyotrophic lateral sclerosis [PATH:ko05014] | -1.10 | 0.01 | 2.47 | Down regulated |
| Apelin signaling pathway [PATH:ko04371] | 2.47 | 0.01 | 2.22 | Up regulated |
| Autophagy - other [PATH:ko04136] | 3.56 | 0.01 | 2.70 | Up regulated |
| Betalain biosynthesis [PATH:ko00965] | -1.30 | 0.02 | 1.82 | Down regulated |
| C-type lectin receptor signaling pathway [PATH:ko04625] | 2.68 | 0.01 | 2.33 | Up regulated |
| Caffeine metabolism [PATH:ko00232] | 3.62 | 0.01 | 2.48 | Up regulated |
| Cell cycle [PATH:ko04110] | -5.38 | 0.05 | 1.31 | Down regulated |
| Chemical carcinogenesis [PATH:ko05204] | -1.13 | 0.05 | 1.32 | Down regulated |
| Chemokine signaling pathway [PATH:ko04062] | 3.38 | 0.02 | 1.77 | Up regulated |
| Chlorocyclohexane and chlorobenzene degradation [PATH:ko00361] | -1.18 | 0.05 | 1.32 | Down regulated |
| Cortisol synthesis and secretion [PATH:ko04927] | 5.00 | 0.01 | 2.28 | Up regulated |
| Endocrine resistance [PATH:ko01522] | 1.72 | 0.04 | 1.42 | Up regulated |
| Ethylbenzene degradation [PATH:ko00642] | -2.03 | 0.05 | 1.33 | Down regulated |
| Fanconi anemia pathway [PATH:ko03460] | -1.67 | 0.03 | 1.54 | Down regulated |
| Fluorobenzoate degradation [PATH:ko00364] | -1.14 | 0.101 | 2.26 | Down regulated |
| Furfural degradation [PATH:ko00365] | -1.46 | 0.01 | 2.00 | Down regulated |
| Gap junction [PATH:ko04540] | 2.25 | 0.03 | 1.57 | Up regulated |
| Geraniol degradation [PATH:ko00281] | -1.35 | 0.02 | 1.75 | Down regulated |
| GnRH secretion [PATH:ko04929] | 29.91 | 0.01 | 2.27 | Up regulated |
| GnRH signaling pathway [PATH:ko04912] | 2.57 | 0.01 | 1.93 | Up regulated |
| Hedgehog signaling pathway - fly [PATH:ko04341] | 3.34 | 0.01 | 2.10 | Up regulated |
| Inflammatory mediator regulation of TRP channels [PATH:ko04750] | 5.00 | 0.01 | 2.28 | Up regulated |
| JAK-STAT signaling pathway [PATH:ko04630] | 3.65 | 0.01 | 2.55 | Up regulated |
| Long-term depression [PATH:ko04730] | 30.66 | 0.01 | 2.52 | Up regulated |
| Long-term potentiation [PATH:ko04720] | 5.23 | 0.01 | 2.69 | Up regulated |
| Measles [PATH:ko05162] | 1.36 | 0.03 | 1.48 | Up regulated |
| Metabolism of xenobiotics by cytochrome P450 [PATH:ko00980] | -1.05 | 0.02 | 1.69 | Down regulated |
| Mitophagy - yeast [PATH:ko04139] | 3.68 | 0.01 | 2.84 | Up regulated |
| Olfactory transduction [PATH:ko04740] | -4.75 | 0.03 | 1.52 | Down regulated |
| Other types of O-glycan biosynthesis [PATH:ko00514] | 2.09 | 0.09 | 2.95 | Up regulated |
| Pathways of neurodegeneration - multiple diseases [PATH:ko05022] | -1.09 | 0.01 | 2.43 | Down regulated |
| Phototransduction - fly [PATH:ko04745] | 28.91 | 0.01 | 2.27 | Up regulated |
| Platelet activation [PATH:ko04611] | 2.76 | 0.01 | 2.45 | Up regulated |
| Prion disease [PATH:ko05020] | -2.20 | 0.01 | 1.59 | Down regulated |
| Proteoglycans [BR:ko00535] | 4.43 | 0.03 | 1.46 | Up regulated |
| Retrograde endocannabinoid signaling [PATH:ko04723] | -2.62 | 0.03 | 1.51 | Down regulated |
| Shigellosis [PATH:ko05131] | -1.01 | 0.03 | 1.76 | Down regulated |
| Styrene degradation [PATH:ko00643] | -1.49 | 0.02 | 1.84 | Down regulated |
| Taste transduction [PATH:ko04742] | 4.72 | 0.01 | 1.78 | Up regulated |
| Tight junction [PATH:ko04530] | 3.77 | 0.01 | 2.03 | Up regulated |
| Toluene degradation [PATH:ko00623] | -1.90 | 0.04 | 1.45 | Down regulated |
| Ubiquitin mediated proteolysis [PATH:ko04120] | -4.14 | 0.03 | 1.49 | Down regulated |
| Wnt signaling pathway [PATH:ko04310] | 2.40 | 0.03 | 1.60 | Up regulated |
| Yersinia infection [PATH:ko05135] | -1.65 | 0.01 | 2.12 | Down regulated |


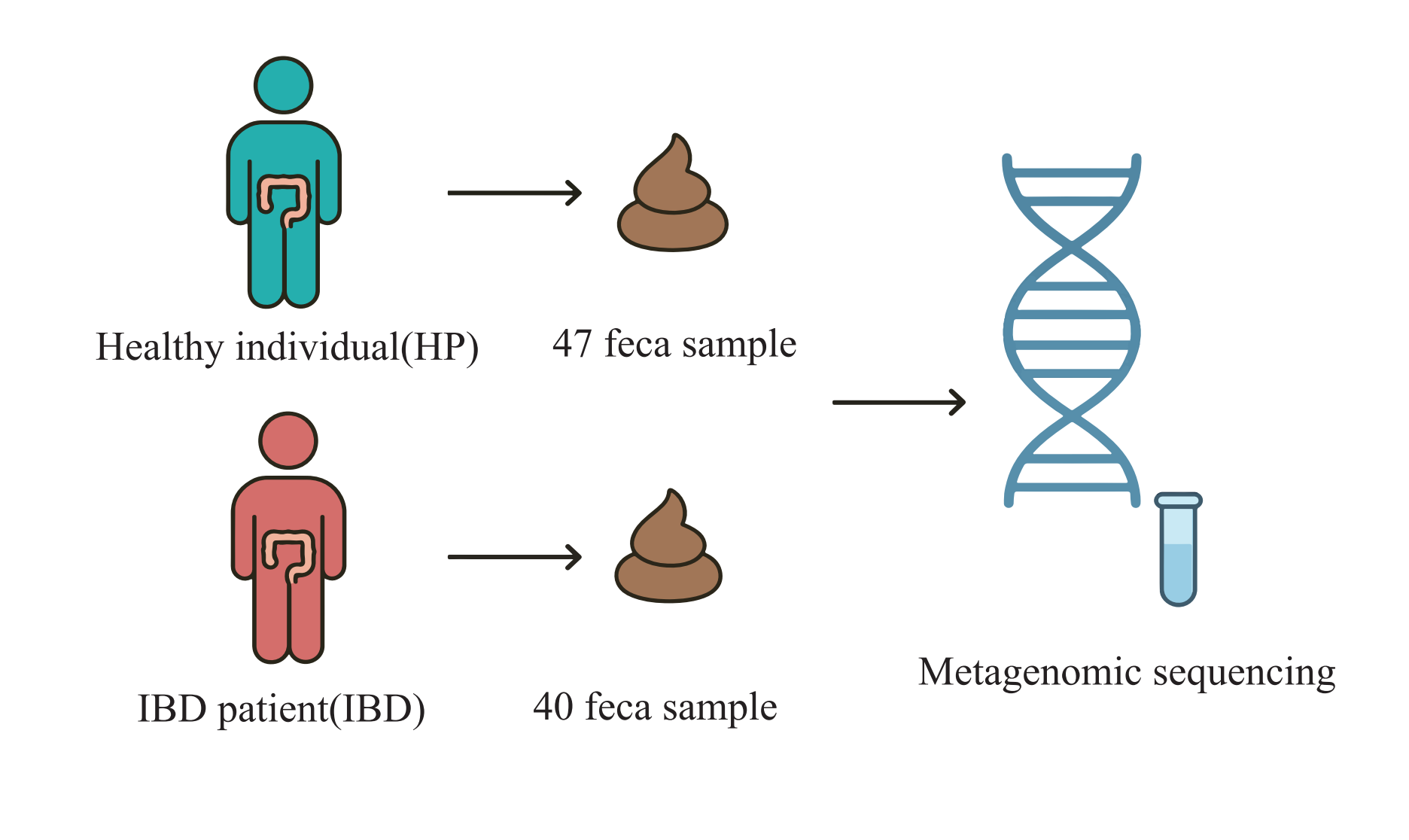
 Figure S1. Schematic overview of the experimental design and study cohort.


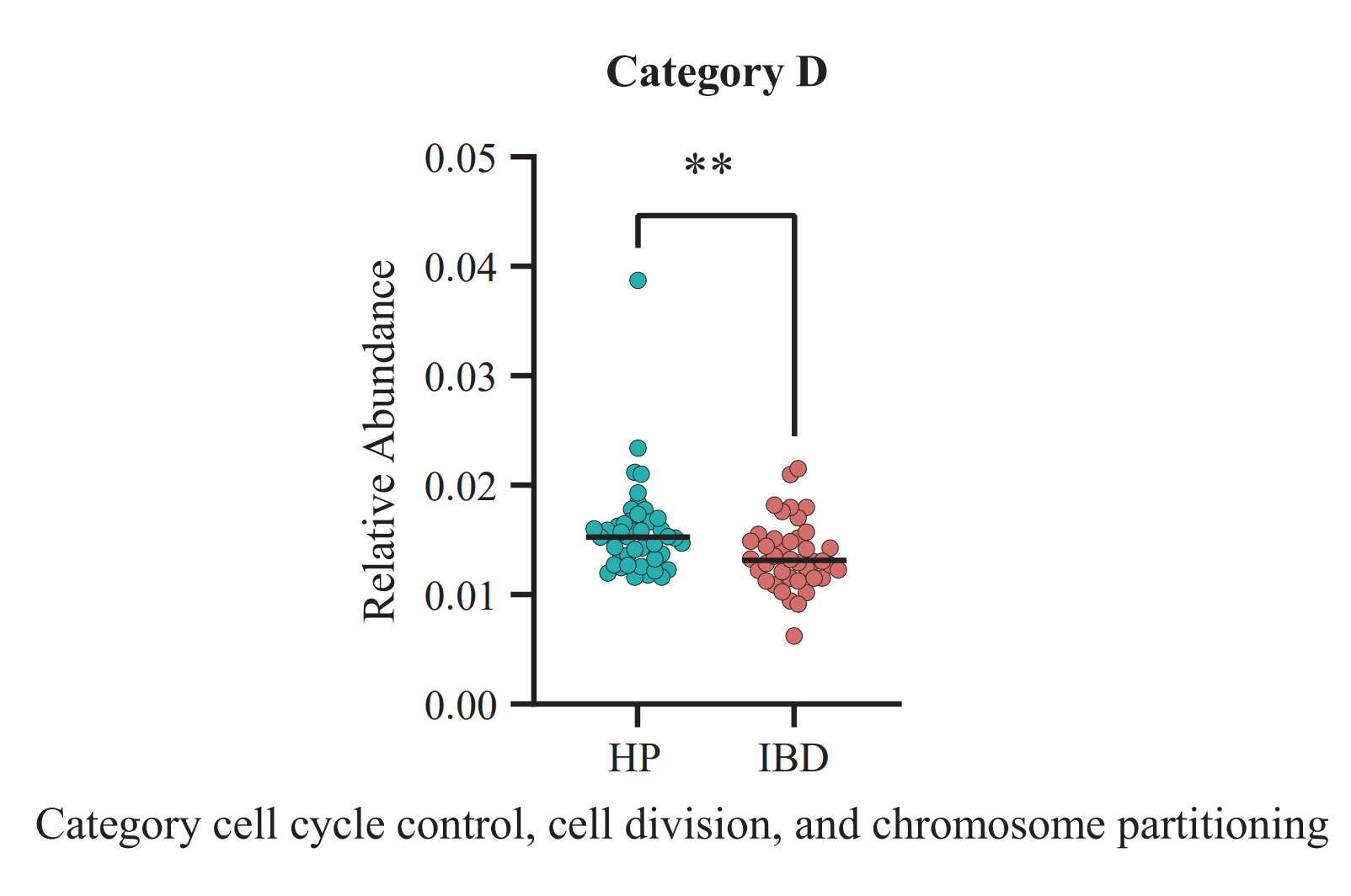


Figure S2. Box plots of differentially abundant COG categories. Red dots represent IBD patients, while blue dots represent healthy individuals.


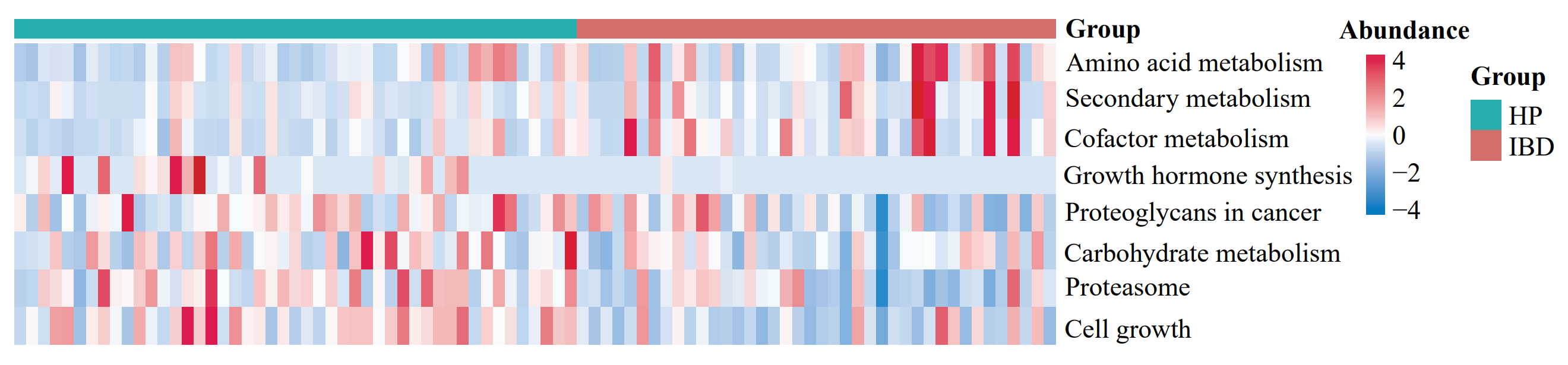


Figure S3. Heatmap of KEGG pathway abundances at level 2. Red indicates a positive correlation, while blue indicates a negative correlation.


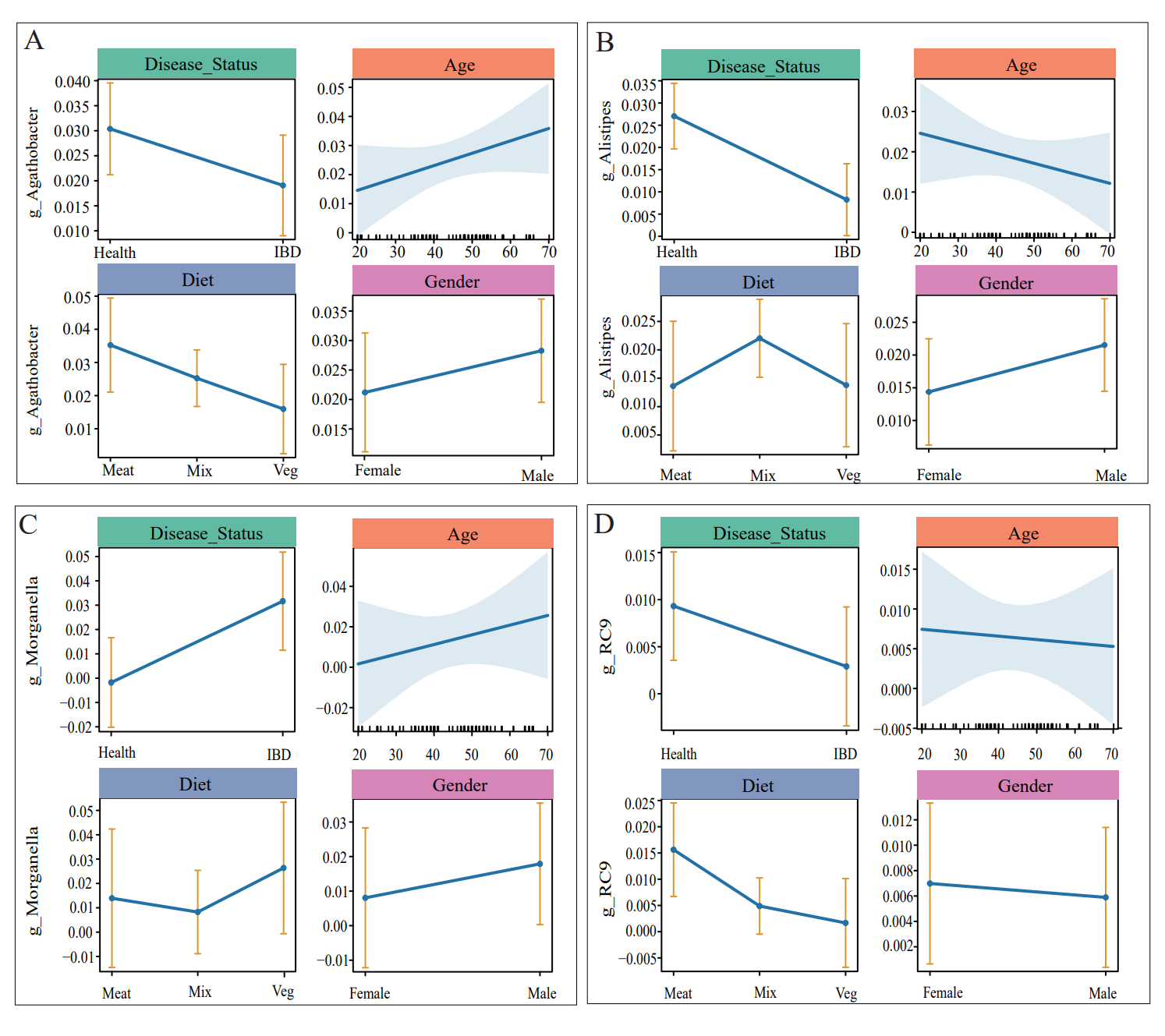


Figure S4. Effect plots from the linear mixed-effects model for selected genera. Panel A shows the model for genus *g_Agathobacter*, panel B for *g_Alistipes*, panel C for *g_Morganella*, and panel D for *g_RC9*.
